# Accurate Driver Detection Exploiting Invariant Characteristics of Smartphone Sensors

**DOI:** 10.3390/s19112643

**Published:** 2019-06-11

**Authors:** DaeHan Ahn, Homin Park, Kyoosik Shin, Taejoon Park

**Affiliations:** 1 Department of Robotics Engineering, Hanyang University, 55 Hanyang-Daehakro, Ansan-si 15588, Gyeonggi-do, Korea; daehani4@gmail.com (D.A.); norwalk87@hanyang.ac.kr (K.S.); 2 BIGHEART, National University of Singapore, MD6, 13 Medical Drive #14-01, Singapore 117599, Singapore; bighp@nus.edu.sg

**Keywords:** driver detection, invariant sensory characteristics, built-in smartphone sensors, distracted driving, driving while distracted

## Abstract

Distracted driving jeopardizes the safety of the driver and others. Numerous solutions have been proposed to prevent distracted driving, but the number of related accidents has not decreased. Such a deficiency comes from fragile system designs where drivers are detected exploiting sensory features from strictly controlled vehicle-riding actions and unreliable driving events. We propose a system called ADDICT (Accurate Driver Detection exploiting Invariant Characteristics of smarTphone sensors), which identifies the driver utilizing the inconsistency between gyroscope and magnetometer dynamics and the interplay between electromagnetic field emissions and engine startup vibrations. These features are invariantly observable regardless of smartphone positions and vehicle-riding actions. To evaluate the feasibility of ADDICT, we conducted extensive experiments with four participants and three different vehicles by varying vehicle-riding scenarios. Our evaluation results demonstrated that ADDICT identifies the driver’s smartphone with 89.1% average accuracy for all scenarios and >85% under the extreme scenario, at a marginal cost of battery consumption.

## 1. Introduction

Driving while distracted (DWD) is a grave threat to the safety of the driver and others. Studies conducted by the United States Department of Transportation indicate that nearly 20% of reported vehicle incidents involve distracted drivers who use smartphones while driving [1,2,3]. Considering the risk of distracted driving to public safety, a number of countries banned smartphone use for all drivers [4]. To further enforce the law, smartphone manufacturers and mobile service providers distributed various smartphone apps to reduce or prevent distracted driving. However, there still exists an increasing number of distracted-driver-related incidents due mainly to their limitation of users having to manually identify themselves as the driver. Such an approach is highly unreliable because users may be reluctant to restrict their favorite mobile services or simply forget to do so.

To date, there have been numerous proposals to improve the reliability of DWD prevention services by automatically detecting drivers by exploiting various sensory features found from driving-related events such as running over a pothole [5], wearing seat-belts [6], and entering a vehicle [7]. However, they are ineffective in real-world settings for the following two reasons. First, these systems were designed assuming that users will always behave within predefined norms that would not distort their proposed sensory features. For example, a smartphone must not be touched when starting a vehicle or must be held in a specific location (e.g., trouser pocket). Such restrictions are highly unrealistic because users may take smartphones out to check emails or send messages whenever they want to. Second, the sensory features are not guaranteed to be found in a timely manner, thus failing to protect the user from the very beginning. Although there exist several studies addressing this issue by identifying the driver before the vehicle leaves its parking spot [8,9], they still need to address the first problem.

Considering the shortcomings of existing solutions, we stress the importance of a reliable system design addressing diverse vehicle-riding actions and unexpected events. To meet this need, we propose a system called ADDICT (Accurate Driver Detection exploiting Invariant Characteristics of smarTphone sensors) exploiting two invariantly observable sensory features found when entering and starting a vehicle. First, one has to twist the body when entering a vehicle, and this action causes one’s smartphone to be rotated as well. We may use either gyroscope or magnetometer sensors to estimate how much the smartphone is rotated. But the latter (magentometer) is inevitably affected by electromagnetic field (EMF) interferences as well as actions taken by the user. As such, we observe different values of rotation between two sensors and the difference is significant enough to discriminate rotations made when entering the vehicle from other activities. Second, although one may use subtle EMF fluctuations when starting a vehicle for driver identification [7], it’s applicability is significantly limited because one’s smartphone should remain static while an engine is being turned on. Such a restriction is mandated since the magnitude of the EMF can be easily overridden by small rotational forces applied to the smartphone. In order to tell unique EMF spikes occurring when starting a vehicle, we propose to employ the engine vibrations on startup (between 8~12 Hz) captured from accelerometer readings. According to our observations, such vibration characteristics cannot be easily mimicked by human motions.

For a comprehensive evaluation of ADDICT, we used Android-based smartphones equipped with inertial measurement units (IMUs) to measure the acceleration, angular velocity, and EMF. We employed three different vehicles and four participants to collect sensory features under three different vehicle-riding scenarios (including basic, common, and extreme scenarios). For the basic scenario, participants were guided not to make any unexpected actions and manipulate smartphones after being seated in a vehicle. In the common scenario, participants were instructed to ride the vehicles as usual, thus generating a moderate amount of noise. Lastly, the extreme case allowed participants to make various unexpected actions to produce a significant amount of noise. Our results demonstrated that entering a vehicle was identified with an 88.3 average area under the curve (AUC) for all cases, while determining the seated row (front or rear) was classified with a 93.5 average AUC for all cases. Overall, ADDICT detected the driver with an accuracy of 86.6~96.6% and identified the passengers with 80.0~96.6% accuracy, thus outperforming existing methods, with a marginal increase in the battery consumption.

The rest of this paper is organized as follows. Section 2 details how our proposed system works. Section 3 presents the results of our comprehensive performance evaluation. Finally, the paper concludes with Section 4.

## 2. The Proposed System

### 2.1. System Overview

When designing ADDICT, we assumed the following. First, users may carry their smartphones in their pockets, bags, or hands while entering the vehicle. Second, If a user places his/her bag, carrying a smartphone, in the rear seats before entering a vehicle, we cannot make accurate decisions. However, we can safely ignore such a case since drivers will not be able to manipulate the phone in the back seat. Lastly, remote vehicle starters are not considered in this paper since most vehicles are not equipped with such an option [9].

**Figure 1:**
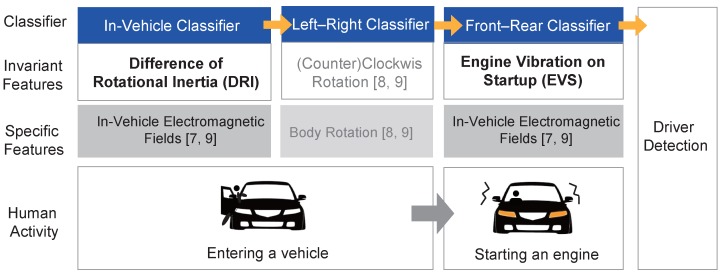
Overview of how Accurate Driver Detection exploiting Invariant Characteristics of smarTphone sensors (ADDICT) works.

As shown in Figure 1, ADDICT consists of an in-vehicle classifier (IVC), a left–right classifier (LRC), and a front–rear classifier (FRC). Considering the fact that vehicle-riding actions are always preceded by walking to and standing by a vehicle, we identify the moment when the user is about to enter the vehicle using accelerometer values as employed in [7,9,10,11]. If the user is found to be standing by the vehicle, the IVC is triggered to determine whether or not the user enters the vehicle by monitoring upcoming user actions. The IVC hinges on an invariant feature, a difference of rotational inertia (*DRI*), from gyroscope and magnetometer readings (see Section 2.2 for details).

Once the IVC confirms that the user has entered the vehicle, the LRC is initiated to determine the boarding side, left or right. Our analyses show that the curvature of the moving trajectory monitored when entering the vehicle from the left side differs from the opposite. The former (left-side entrance) generates a clockwise curve while the latter produces a counterclockwise motion. The LRC combines both accelerometer and gyroscope readings at the moment of vehicle entry to compute the moving trajectory and determines whether or not the trajectory corresponds to the clockwise curvature using, e.g., a fuzzy inference system as presented in [8].

After identifying the boarding-side, the FRC is triggered to differentiate seated rows, front or rear, using an invariant feature produced when the engine is turned on. According to [7,9], starting the vehicle causes a subtle in-vehicle EMF fluctuation with a relatively greater magnitude around the driver’s seat than the rear seats. However, manipulating the smartphone also causes EMF readings to fluctuate, causing false alarms by classifying the user seated in the rear as being in the front. To overcome this problem, we exploit engine vibrations monitored by the accelerometer when starting a vehicle, which cannot be easily generated by the users. By using the engine vibrations on startup (*EVS*), FRC achieves accurate classification results (see Section 2.3 for details).

### 2.2. Difference of Rotational Inertia

**Figure 2:**
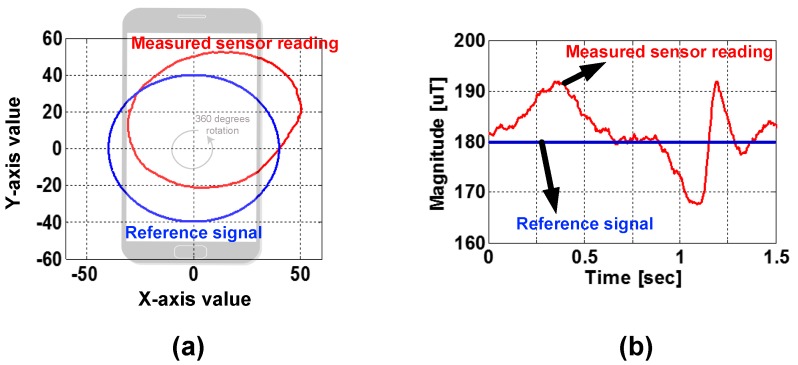
Discrepancy between measured and reference values of magnetometers: (**a**) magnetometer’s (x,y) values while a smartphone is rotated by 360${}^{\circ }$, and (**b**) magnetometer readings in the time domain.

To detect the moment of vehicle entry, one may use EMF values of magnetometers induced by the vehicle [7,9]. But unfortunately, similar EMF features can be monitored from other activities of users holding smartphones. For instance, similar EMF fluctuations may occur with a simple motion such as raising and lowering the smartphone. Figure 2a depicts a plot of a magnetometer’s (x,y) values while a smartphone is rotated by 360${}^{\circ }$. Ideally, it forms a circle with an origin at (0,0) and completely overlaps with a reference signal. But in reality, it moves away from the reference signal, which contributes to distortions in sensing accurate EMF values. Moreover, measured EMF values fluctuate when the smartphone is rotated as shown in Figure 2b. This means existing solutions based on EMF fluctuations suffer from frequent false alarms [12,13].

**Figure 3:**
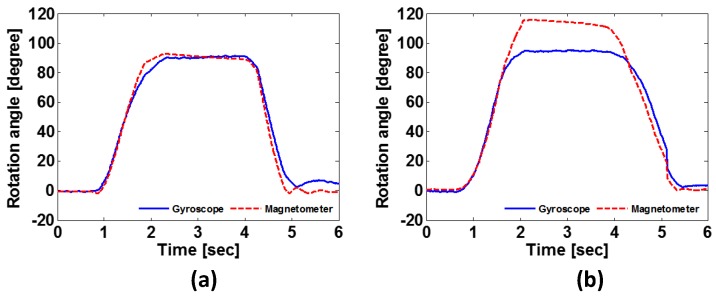
How the rotation angles of the magnetometer and accelerometer vary when the smartphone is turned by 90${}^{\circ }$: when (**a**) there is no electrical interference, and (**b**) electrical interferences exist.

To address this problem, we exploit the fact that rotation angles individually estimated from the gyroscope and magnetometer do not match well if there exist EMF interferences induced by the vehicle, leading to an invariant feature, the difference of rotational inertia (*DRI*). A magnetometer has three axes (*x*, *y*, and *z*), each of which varies proportionally with the rotation of a smartphone (magnetometer) as well as the strength of surrounding magnetic fields. In fact, Figure 3 plots how rotation angles of both sensors vary when the smartphone is rotated 90${}^{\circ }$. Figure 3a shows that the readings vary from 0${}^{\circ }$ to 90${}^{\circ }$ under no EMF interferences. In this case, both sensors give similar values that correspond to the tilt of a smartphone. By contrast, as shown in Figure 3b, at 113 s the two sensors produce different results when there are EMF interferences, i.e., the output of the magnetometer is larger than that of the gyroscope since the former accumulates the effects of in-vehicle EMF as well. Thus, we translate this difference into an amount of in-vehicle EMF in terms of *DRI*, which is calculated by:

(1) $$DRI=max\left|\theta_{mag}^{\prime }\left(t\right)-\theta_{gyr}^{\prime }\left(t\right)\right|,$$

where $\theta_{mag}^{\prime }\left(t\right)$ and $\theta_{gyr}^{\prime }\left(t\right)$ are the sums of integrated (x,y,z) readings of the magnetometer and the gyroscope at time *t*, respectively, capturing the maximum difference between the magnetometer and gyroscope while the user is sitting down. With an appropriately chosen cut-off threshold, the *DRI* assumes that the user has entered the vehicle if the difference exceeds the threshold.

### 2.3. Engine Vibrations on Startup

The FRC aims to identify whether the smartphone is located at the front or the rear, even when there exist various unexpected activities inducing unwanted noise. Previous studies pointed out that the EMF spike monitored at the front row is relatively stronger than that of the rear when starting the vehicle [7,9]. However, solely relying on the EMF feature introduced by previous solutions suffers from a high risk of false alarms in real-world scenarios because human activities such as door closing or taking a smartphone out of a bag may also incur EMF variations, as described in Section 2.2. We therefore aim to reinforce the FRC with the capability of identifying whether or not the EMF changes are caused by the engine startup.

**Figure 4:**
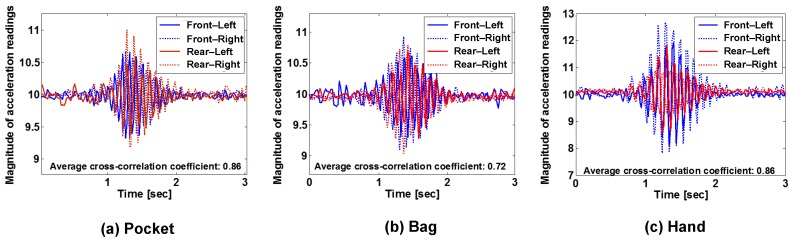
Variations in the magnitude of acceleration during the startup of engines when smartphones are held in: (**a**) pockets, (**b**) bags, and (**c**) hands.

It is important to employ a new feature for engine startup that appears regardless of the smartphone’s position. After monitoring various sensor readings by varying smartphone positions and types of vehicles, we find the accelerometer is suitable for sensing the *EVS*. Figure 4 plots variations in the magnitude of acceleration during the startup of engines. From the figure, all of the magnitude changes follow similar patterns, i.e., lasting around 1 s of vibration, experiencing a slight increase after the engine startup, and then gradually decreasing over time. We use a correlation coefficient (for each smartphone position) to quantify the similarity of *EVS* among different seat positions. The average correlation coefficients are computed as 0.86, 0.72, and 0.86 for the cases of smartphones held in pockets, bags, and hands, respectively. This means *EVS* is highly correlated with smartphone location since the coefficients are larger than a threshold of 0.6 [14,15]. In addition, we compute the similarity of smartphone positions in terms of cross-correlation coefficients (mean ± standard deviation) as follows:

- cross_correlation(pockets,hands)=0.92±0.02,
- cross_correlation(hands,bags)=0.83±0.13, and
- cross_correlation(bags,pockets)=0.79±0.19.

This means *EVS* has little to do with the smartphone positions. Finally, we extract *EVS* patterns from four vehicles to yield an average cross-correlation coefficient of 0.71. All of these results indicate that *EVS* can be used to reliably detect when the engine starts, regardless of seat position and vehicle types.

**Figure 5:**
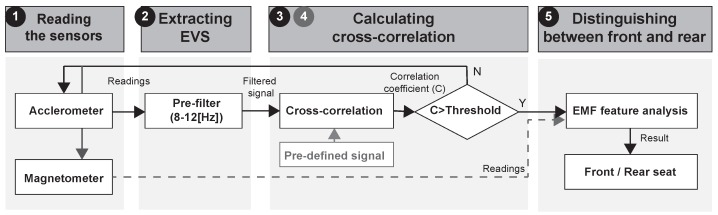
The workflow of the front–rear classifier (FRC). EMF—electromagnetic field.

To deal with situations of accelerometer readings being distorted by human activities during engine startup, we extract and use frequency features in the range of 8~12 Hz. Accordingly, as shown in Figure 5, the FRC executes the following steps:

1. It reads accelerometer and magnetometer readings for a predefined duration of time, which is set to 2 s in our experiments.
2. It passes the accelerometer readings through a pre-filter to extract *EVS* within a frequency band of 8~12 Hz while removing interferences and noises.
3. It determines the similarity between the filtered *EVS* and the reference pattern by calculating a cross-correlation coefficient.
4. If the similarity is less than 0.6, it goes back to step 1.
5. Otherwise, it analyzes the magnitude of the EMF to distinguish between the front and rear.

**Figure 6:**
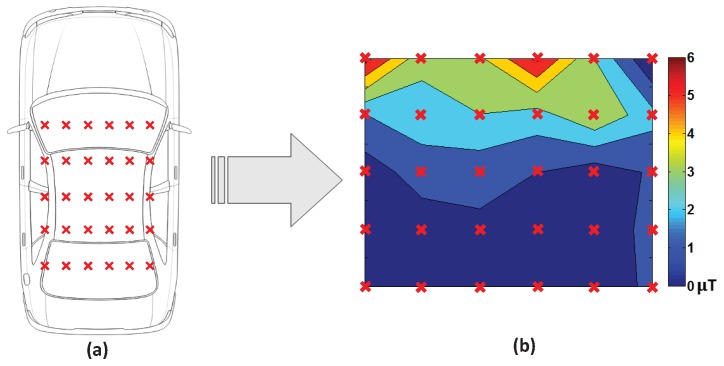
Experiments on EMF changes: (**a**) measurement locations in a vehicle, and (**b**) sample variances of EMF values during engine startup.

As mentioned earlier, the magnitude of EMF changes at the front seat is larger than the rear seat. To quantify the EMF change, we employ a sample variance of EMF values, as visualized in Figure 6. We observe that computed values near the front (rear) seat are larger (smaller) than 1 μT. Finally, like the IVC, the FRC uses the Bayesian classifier to determine the seated row, minimizing the error rate. We set a threshold $\tau_{frc}$ to 0.98 to distinguish between the front and rear seats.

## 3. Performance Evaluation

### 3.1. Experimental Setup

We used Android-based smartphones (Galaxy S5, Samsung Electronics, Seoul, Korea), each with a built-in inertial measurement unit (IMU) to measure the acceleration, angular velocity, and EMF. The sampling rate of the IMU was set to 20 ms. We employed four participants who consented to participate in our study after receiving detailed information on the procedures, scenarios, and potential risks. Note that our participants were in their mid 20s and early 30s, as shown in Table 1, and hence we did not consider the behavioral patterns of elderly and disabled individuals who might have limited physical movements. Furthermore, wild actions likely to be found in kids were also omitted from our design since they are not allowed to drive on a public road. The participants repeatedly rode three vehicles from three different segments widely found in our daily lives. The vehicles included a Hyundai Accent, Kia K5, and Hyundai Grandeur from the subcompact, mid-sized, and large-sized segments, respectively. While other vehicle types such as trucks and sport utility vehicles are not considered in this work, we stress that ADDICT exploits invariant sensory features found in vehicles running on petrol engines.

The participants were instructed to stand at least 20 m away from the vehicles to include walking, standing, and vehicle-riding actions. In addition, participants carried their smartphones in three different positions (pockets, bags, and hands) and behaved according to the following scenarios.

**Basic** **scenario:** participants did not manipulate their smartphones when entering, thus incurring only three events (standing, sitting, and engine-starting).
**Common** **scenario:** participants were allowed to use their smartphones while entering a vehicle (e.g., texting and phone call), which generates noise that could distort/override required sensory features.
**Extreme** **scenario:** participants were allowed to perform unexpected actions (e.g., swinging while walking, and shaking) that are less likely to be observed during vehicle-riding events, to produce a significant amount of noise.

We employed both receiver operating characteristic (ROC) curves and area under the curve (AUC) values to evaluate the performance of the IVC and FRC and demonstrate their feasibility against other classifiers proposed in [9]. The ROC curve illustrates the performance of a binary classifier where the curve is generated with the true positive rate against the false positive rate under various threshold settings. The performance of a classifier can also be evaluated by computing the AUC, where an AUC close to 1 indicates that the system is able to differentiate vehicle entry perfectly from other activities while an AUC≤0.5 indicates that the classifier is meaningless.

### 3.2. Performance of In-Vehicle Classification

To evaluate the performance of the IVC in differentiating the vehicle-riding actions from other daily activities involving similar sitting motions, we conducted two separate experiments where the four participants recorded their (1) vehicle-riding actions and (2) sitting-down motions in front of an office desk (with non-swivel and non-rotating chairs), following the three scenarios we defined previously. The office environment was carefully selected among others because electronic devices found around the office desk emit EMFs that likely distort the electromagnetic sensor readings. For each experimental condition (a total of 6 experimental conditions as shown in Table 2), each participant recorded 62 trials, giving us a total of 1488 data instances.

**Figure 7:**
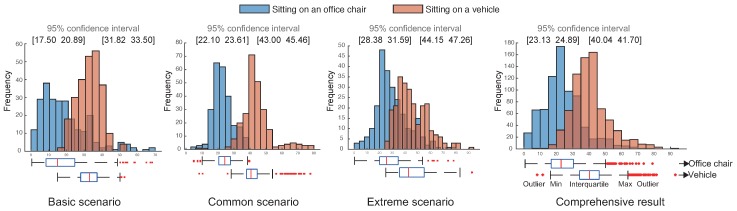
Histograms of *DRI* values in two cases: vehicle entry and sitting in daily living.

Since the characteristics of each distribution representing six different experimental conditions shown in Table 2 were unknown, we first conducted the normality test called the Shapiro–Wilk W test [16] to validate whether these distributions, shown in Figure 7, follow the Gaussian behavior. The test results in Table 2 indicate that most of the distributions did not follow the Gaussian behavior, except for the vehicle-riding actions under the basic scenario.

Therefore, we further investigated the distinction between vehicle-riding actions and sitting-on-an-office-chair motions for each scenario, using a non-parametric test called the Mann–Whitney U test [17]. The results in Table 2 illustrate that there was a less than 0.01% risk of concluding that a difference exists for all combinations of distributions when there was no actual difference. In other words, the distributions found from vehicle-riding and sitting-on-an-office-chair actions were statistically different and thus could be safely and successfully differentiated. We applied a logistic regression model [18] to differentiate vehicle entry from the sitting-down motion.

**Figure 8:**
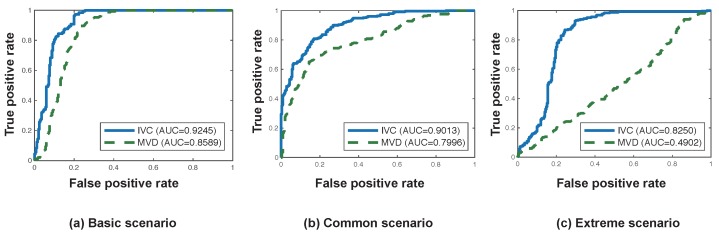
Receiver operating characteristic (ROC) curves and area under the curve (AUC) values of the in-vehicle classifier (IVC) and magnetic field variance detection (MVD) under three (basic, common, and extreme) scenarios.

The performance of the IVC is further evaluated against the magnetic field variance detection (MVD) [7,9]. Figure 8 shows the ROC curves and AUC values of the IVC and MVD under three scenarios. As expected, MVD had AUCs of 0.85, 0.79, and 0.49 in the basic, common, and extreme scenarios, respectively. This means that MVD could not differentiate vehicle-riding-actions and non-vehicle-related sitting actions. In contrast, the IVC achieved AUCs of 0.92, 0.90, and 0.82 for the three scenarios, which means that the IVC outperforms MVD and maintains a high performance even in the extreme settings.

### 3.3. Performance of Front-Rear Classification

**Figure 9:**
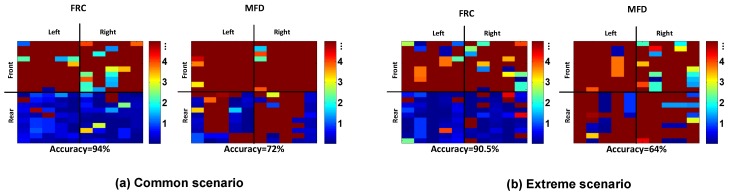
Results of front vs. rear classification in (**a**) the common scenario, and (**b**) the extreme scenario. MFD—magnetic field fluctuation detection.

We evaluated both the FRC and an existing method called MFD (magnetic field fluctuation detection) [7,9], where the latter utilizes EMF fluctuations when starting the vehicle. Both the FRC and MFD showed very similar performances in the basic scenario, and hence we focused on the rest of scenarios. Figure 9 plots the results of the FRC and MFD evaluation. If accurately classifying between the front and rear row, the front row is marked in red and the rear row in blue. In both scenarios, the FRC maintained much clearer separation between the front and rear compared to MFD. While MFD provided an accuracy of 72% and 64%, the FRC achieved 94.0% and 90.5% for common and extreme scenarios, respectively.

**Figure 10:**
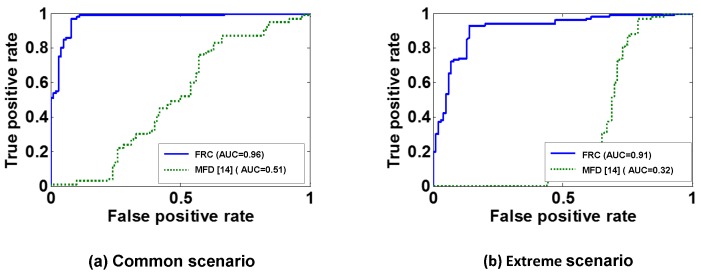
ROC curves and AUC values of the FRC and MFD under the common and extreme scenario.

Figure 10 plots the ROC curves and AUC values of the FRC and MFD. MFD had AUCs of 0.51 and 0.32 in the common and extreme scenarios, respectively, which means that MFD performed poorly in classifying the seated row, agreeing with the results of Figure 9. However, the FRC achieved AUCs of 0.96 and 0.91 for common and extreme scenarios, respectively. This indicates that the FRC is capable of accurately detecting the seated rows in real environments.

### 3.4. Performance of Driver Detection

**Figure 11:**
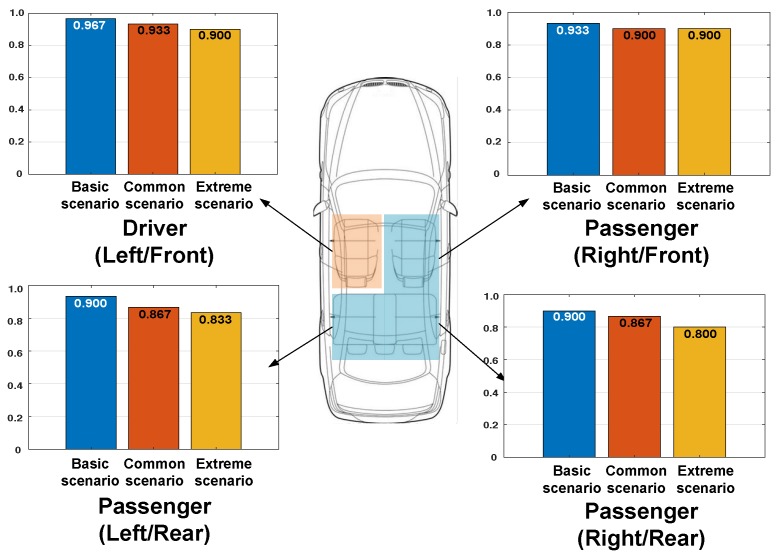
The results of detecting the driver and passengers in three scenarios.

We distributed ADDICT-enabled smartphones to the participants and conducted experiments in three vehicles by varying smartphone positions and scenarios. Figure 11 shows the results of each seat position. ADDICT achieved very high accuracy for detecting the driver, i.e., 96.7%, 93.3%, and 90.0% for the basic, common, and extreme scenarios, respectively. In addition, it accurately detected passengers’ seats with an accuracy of 93.3~90.0%, 90.0~86.7%, and 90.0~80.0% for the three respective scenarios. These results clearly demonstrate that ADDICT maintains a high level of accuracy in distinguishing between the driver and passengers, even in extreme (or real-world) settings.

### 3.5. Energy Consumption

Finally, we evaluated the energy consumption incurred by ADDICT. To do so, we developed an application to record the battery level when all services such as WiFi, GPS, and Bluetooth were turned off and while a display was maintained at the highest level of brightness. Our experimental results indicate that ADDICT shortens the smartphone’s battery lifetime by 132 min. This means that one may use the ADDICT-enabled smartphone for nearly 10 h when the default smartphone covers 12 h of battery usage.

### 3.6. Discussion

While ADDICT demonstrated a significant performance advantage over other state-of-the-art solutions, there are several limitations which need further investigation. First, ADDICT was designed and evaluated using a representative set of vehicle types that all run on petrol engines installed at the front of the vehicles. The EMF interference and engine vibration patterns of vehicles with rear engines (e.g., Porsche 911) and electric vehicles (e.g., Tesla Model S) are yet to be discovered. Furthermore, other vehicle types that could require different vehicle-entering actions, such as trucks, SUVs, buses, and more, are not considered in our study.

Second, it is important to point out that ADDICT (and other works as well) does not always guarantee a perfect result. Our evaluation results showed that drivers are detected with an accuracy of 86% under the extreme scenario. Therefore, DWD prevention services should consider incorporating additional checking mechanisms that exploit various driving-related sensory features introduced by other researchers, in addition to our method. However, we stress the importance of identifying the drivers (or drivers’ smartphones) before the vehicle leaves its parking spot, to ensure the safety of everyone.

## 4. Conclusions

In this work, we presented an accurate and reliable driver detection system called ADDICT that is composed of three classifiers exploiting invariant characteristics extracted from daily vehicle-riding actions. The IVC determined if a user entered a vehicle based on the difference in rotational inertia between the gyroscope and the magnetometer. Once the IVC detected the vehicle entry, the LRC analyzed the curvature of the movement trajectory monitored when entering the vehicle to differentiate the boarding side, left or right. Lastly, the FRC identified the seated row, front or rear, exploiting in-vehicle electromagnetic field fluctuations and engine vibrations. We evaluated ADDICT under three different vehicle-riding scenarios to achieve an overall performance of 92.50%, 89.16%, and 85.83% for the basic, common, and extreme scenarios, respectively, demonstrating superior performance compared to existing methods.

## Figures and Tables

**Table 1:** Participant information.

Subject	Sex	Age	Height	Weight
P1	M	30	179	90
P2	M	31	181	85
P3	M	25	173	80
P4	F	26	160	51

**Table 2:** Characteristics of distributions attained from different experimental conditions.

Condition		Summary Statistics					Shapiro–Wilk W Test		Mann–Whitney U Test
Scenario	Environment	Min	Median	Max	Mean	Std. Dev.	W	Prob < W	*p*-Value
Basic	Office chair	0.76	15.90	69.94	19.19	13.61	0.898	<0.0001	<0.0001
	Vehicle	15.05	33.05	53.98	32.66	6.74	0.989	0.0626	
Common	Office chair	5.03	22.59	38.44	22.85	6.07	0.982	0.0033	<0.0001
	Vehicle	6.78	42.81	79.53	44.23	9.88	0.888	<0.0001	
Extreme	Office chair	2.85	27.56	78.18	29.99	12.89	0.957	<0.0001	<0.0001
	Vehicle	25.41	42.58	93.72	45.71	12.49	0.944	<0.0001	
